# Synthesis of novel nano composite based on silicone/HEMA/AAm/ZnO as an efficient wound dressing: physicochemical and biological study

**DOI:** 10.1080/15685551.2026.2714638

**Published:** 2026-08-06

**Authors:** Atieh Karbalaei, Sana Sadraei-Majd, Mohammad Taghi Khorasani, Mehrdad Behmanesh

**Affiliations:** a Department of Nanobiotechnology, Faculty of Biological Sciences, Tarbiat Modares University, Tehran, Iran; b Department of Biomedical Engineering, Faculty of Chemical Engineering, Tarbiat Modares University, Tehran, Iran; c Department of Biomaterials, Iran Polymer and Petrochemical Institute, Tehran, Iran; d Department of Genetics, Faculty of Biological Sciences, Tarbiat Modares University, Tehran, Iran

**Keywords:** Hydroxyethyl methacrylate, acrylamide, silicone, IPN, wound dressing, nanoparticle

## Abstract

Modern wound dressings are used for burn injuries to prevent complications such as infection and to promote tissue regeneration. In this study, 2-hydroxyethyl methacrylate (HEMA), acrylamide (AAm), and silicone were simultaneously copolymerized in a dioxane solution via an interpenetrating polymer network (IPN) method to produce nanoparticles (NPs). In the next step, to fabricate nanocomposite (NC) films for wound dressing applications, the IPN NPs were blended with silicone and zinc oxide (ZnO) NPs as an antibacterial agent. Transmission electron microscopy (TEM) showed that the synthesized IPN NPs had an average size of approximately 17 nm. Fourier-transform infrared (FTIR) analysis of the samples confirmed the presence of the characteristic functional groups of the NC films. Scanning electron microscopy (SEM) images revealed that the NC films had a homogeneous structure with well-dispersed NPs. Despite the high silicone content, the films exhibited an acceptable swelling ratio (up to 165%). Biological studies using L929 mouse fibroblast cells showed that the samples were biocompatible. In vivo wound-healing studies in a rat wound model specified improved healing compared with the control group, with complete re-epithelialization within 14 days. Histological analysis indicated neovascularization and tissue regeneration. Overall, the synthesized NC films show potential as wound dressings.

## 1. Introduction

Wound healing requires dressings that provide moisture balance, mechanical protection, and antimicrobial activity. Silicone-based films and interpenetrating polymer network (IPN) systems have been widely studied for this purpose. However, most previous designs rely on bulk polymer blends or direct IPN formation within the final film. These approaches frequently suffer from phase separation, limited nanoscale control, and suboptimal hydration-mechanical balance [1,2].

To address this limitation, various strategies have been proposed, including nanocomposite [3], double-network [4], and interpenetrating polymer networks (IPN) systems [5]. Incorporating synthetic or natural polymers into an IPN hydrogel film can enhance mechanical strength and create synergistic interactions among the components [6,7]. Accordingly, IPN formation has been widely reported as an effective approach to improve polymer performance without synthesizing entirely new polymer chemistries [8–10]. Similarly, nanocomposite (NC) films can provide unique chemical and physical properties [11]. Beyond the direct use of nanoparticles (NPs) as therapeutic agents for wound healing, nanoscale polymeric systems have attracted considerable interest because of their high surface area and their capacity to load bioactive compounds on the particle surface [12].

Among available polymers, silicone, particularly room-temperature vulcanized (RTV) silicone, is frequently used in clinical applications due to its biocompatibility and excellent mechanical properties [13]. Silicone-based dressings are commonly used for skin wound care because they establish proper contact with the wound bed and surrounding skin [14]. Their low adhesion to the tissue can reduce trauma during dressing changes, support cell growth, and ultimately accelerate wound healing, while reducing the patient's pain [15]. RTV-2 silicone is particularly suitable for constructing interpenetrating polymer networks (IPNs) because it contains terminal vinyl groups that can participate in covalent crosslinking reactions. When combined with a TEGDEMA-crosslinked hydrophilic network, these reactive vinyl functionalities enable chemical bonding between the silicone matrix and the hydrogel phase [16,17]. This compatibility ensures the formation of a true IPN architecture, providing enhanced mechanical stability, promoting a more uniform dispersion of hydrophilic domains, and improved water absorption compared with systems based primarily on physical entanglement [18].

The incorporation of hydrophilic polymers such as 2-hydroxyethyl methacrylate (HEMA) and acrylamide (AAm) can effectively compensate for the water absorption properties of silicone-based materials [19]. AAm is widely used in hydrophilic polymer networks because of its high water absorption capacity [20]. In addition, the presence of amide groups provides reactive sites that contribute to the chemical functionality of AAm-based materials [21]. Previous studies have also reported that AAm-based substrates can exhibit antibacterial activity and good hemocompatibility, making them attractive for applications such as drug delivery systems [22]. HEMA is likewise widely used in the biomaterials field due to its excellent mechanical properties, biocompatibility, and non-toxicity [23,24], and it has been applied in contact lenses, wound dressings, and drug delivery systems [25].

Infection is a major concern in burn wounds and can significantly delay healing [26]. Therefore, incorporating antibacterial agents into wound dressings is essential to reduce infection risk and accelerate wound repair [27]. Among the nanoparticles used in wound dressing applications, zinc oxide (ZnO) nanoparticles have received considerable attention [28]. ZnO NPs are readily available, biocompatible, inexpensive, and non-toxic, and are therefore widely used in biomedical applications [29]. In addition to their antibacterial and anti-inflammatory properties, ZnO NPs have been reported to promote re-epithelialization, which accelerates the wound healing process [30–32].

The purpose of this study is to fabricate silicone-based nanocomposite films and evaluate their physicochemical properties for wound dressing applications. In this work, we introduce a novel design strategy based on IPN nanoparticles, which are synthesized as copolymer nanostructures and subsequently incorporated into a silicone matrix with ZnO nanoparticles. The key innovation of this study lies in using these pre-formed IPN nanoparticles as nanoscale building blocks that create a dual-network architecture inside the silicone/ZnO matrix. This design enables superior compatibility between hydrophilic and hydrophobic phases, uniform nanoparticle dispersion, enhanced mechanical integrity, and balanced water absorption capacity compared to conventional bulk silicone/poly(HEMA-co-AAm) blends or silicone-ZnO composites [33,34]. By combining the advantages of IPN nanotechnology with silicone and ZnO in a film, the present system offers a distinct architecture that addresses the limitation of previously reported materials while providing promising physiochemical and biological performance for advanced wound dressing applications.

## 2. Materials and methods

### 2.1. Materials

Acrylamide, 2-Hydroxyethyl methacrylate, triethyleneglycol dimethacrylate (TEGDMA), 1,4-dioxane, and 1,3-Phenylene-bis-oxazoline (1,3-PBO) were obtained from Merck. Room-temperature vulcanizing silicone rubber (RTV-2 silicone; Sylgard 184) was obtained from Dow Corning. Mouse fibroblast cells (L-929) were purchased from the Pasteur Institute of Iran. Trypsin-EDTA, Dulbecco's Modified Eagle's Medium/Nutrient Mixture (DMEM/F12) solution, and fetal bovine serum (FBS) were purchased from Gibco, Germany. Escherichia coli (*E. coli*) and Staphylococcus aureus (S. aureus) bacteria were provided by the Royan Institute of Iran. Ethanol and dimethyl sulfoxide (DMSO) were supplied by Merck.

### 2.2. Synthesis of poly (AAM-co-HEMA)/silicone IPN nanoparticles

Poly (AAM-co-HEMA)/silicone IPN powders were synthesized at four ratios using an IPN polymerization approach [35], as described in Table 1. The monomers were dissolved in 1,4-dioxane solvent to prepare a 5 wt% solution. TEGDMA (0.1 wt%) was used as the crosslinker for the hydrophilic copolymer network, and 1,3-PBO (7.5 × 10^−3^) was added as a coupling agent. Polymerization occurred at 70 °C for 10 hours under a nitrogen atmosphere. The resulting product was freeze-dried for 48 hours to remove the solvent and obtain the IPN powder, yielding approximately 90%. Notably, the poly(AAM-co-HEMA)/silicone system forms a physically interpenetrated IPN, in which simultaneous network formation and condensation of silicone enable strong non-covalent coupling (hydrogen bonding and van der Waals interactions) without direct covalent linkages, thus distinguishing it from a simple semi-IPN.

**Table 1.** The composition of poly (AAM-co-HEMA)/silicone IPN nanoparticles.

Sample name	AM (g)	HEMA (cc)	Silicone (g)
IPN-75% silicone	1	0.25	3.75
IPN-50% silicone	2	0.5	2.5
IPN-25% silicone	3	0.75	1.25
IPN-10% silicone	3.5	1	0.5

### 2.3. Preparation of nanocomposite films

**Scheme 1.**
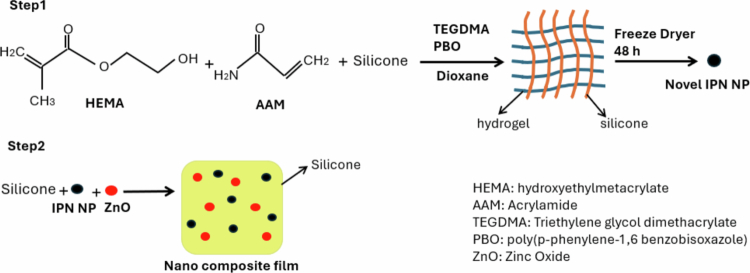
Step 1 shows the poly (HEMA-co-AAm)/silicone/IPN NPs synthesis method. Step 2 shows the preparation of the NC film.

To prepare the silicone-based NC films, 1 g of the IPN nanoparticle (Table 1) and 0.2 g of ZnO NPs were added to 1 g of silicone in the corresponding container (Table 2). The components were then mixed using a homogenizer, poured into Petri dishes, and cured at room temperature to obtain NC films (as shown in Scheme 1). As a result, four film formulations containing different IPN nanoparticle compositions were prepared.

**Table 2.** The composition of NC films.

Sample (films)	Silicone (g)	Nanoparticle (IPN 75%) (g)	Nanoparticle (IPN 50%) (g)	Nanoparticle (IPN 25%) (g)	Nanoparticle (IPN 10%) (g)	ZnO (g)
NC1	1	1	-	-	-	0.2
NC2	1	-	1	-	-	0.2
NC3	1	-	-	1	-	0.2
NC4	1	-	-	-	1	0.2

### 2.4. Attenuated total reflection Fourier transform infrared (ATR-FTIR) spectroscopy

ATR-FTIR spectroscopy was performed on the pristine materials and all NC film samples using an Equinox 55 spectrometer equipped with an ATR accessory. Spectra were collected in the range of 4000–650 cm^−1^ with a resolution of 2 cm^−1^.

### 2.5. Morphology

The morphology of nanocomposite films was examined using a scanning electron microscope (SEM) (Vegall, Tescan). Transmission electron microscopy (TEM) was used to determine the particle size (Philips Electron Optics, Netherlands). Elemental composition was analyzed using energy-dispersive X-ray spectroscopy (EDX) coupled to SEM, and both qualitative and quantitative analyzes were conducted on the samples. All measurements were performed at 25 °C.

### 2.6. Examining physical and mechanical properties

The tensile test was performed for the NC films using the Zwick/Materialprüfung (ZwickCo., Germany) with a 10 N load cell and a deformation rate of 10 mm/min. The samples were tested with dimensions of 50 mm × 10 mm × 0.2 mm. The tensile modulus of each sample was determined from the initial linear region of the stress–strain curve [35].

### 2.7. Hydrophilicity

The water contact angle was measured by a contact angle goniometer (G10-Kruss, Germany). The NC films were cut into 30 × 10 mm pieces and placed on the movable stage of the apparatus [36]. Then, a drop of distilled water, about 3–5 μL, was placed on the film surface using a micro-syringe [37,38].

### 2.8. Gel content

To measure gel content, lyophilized samples were first weighed (W_d_) and then immersed in buffer solution at 37 °C for 2 days. The buffer solution was replaced every 12 hours to remove soluble fractions. Afterward, the samples were lyophilized for 24 hours until a constant weight was obtained (W_g_) [39]. The gel content is calculated using the following Equation (1):

(1) $$\mathrm{Gel}\;\mathrm{content}=(W_{g}/W_{d})\times 100\;$$

### 2.9. Swelling ratio

To measure the swelling ratio, samples were cut into squares (2 cm × 2 cm) and dried at room temperature for 24 hours. The dried samples were weighed and immersed in 25 mL of distilled water for 72 hours. The swelling ratio is calculated from the following Equation (2):

(2) $$\mathrm{Swelling}\;\mathrm{ratio}=(W_{s}-W_{d})/W_{d}$$

Where W_s_ and W_d_ are the weights of swollen and dry samples, respectively.

### 2.10. Water vapor transmission rate (WVTR)

The water vapor transmission rate (WVTR) was calculated according to the European Pharmacopoeia (EP) standard [40]. All NC films (0.2 mm thick, 35 mm diameter) were sealed over bottles with a 34 mm mouth diameter containing 25 mL distilled water using Teflon tape. The bottles were incubated at 37 °C for 24 hours, and then reweighed [41]. WVTR was obtained from the following Equation (3):

(3) $$\mathrm{WVTR}(gm^{2}d^{-1})=(\mathrm{Wi–Wf})/A$$

Where A is the permeation area of samples, and W_i_ and W_f_ are the initial and final weights of bottles, respectively.

### 2.11. Cell viability

The cell viability of all NC films was assessed based on the viability of mouse fibroblast cells (L-929) according to the ISO 10993-5 standard. According to the ISO standard, the extracts of the samples were prepared in a culture medium (DMEM/10% FBS/1% antibiotics) for 24 and 72 hours. Optical density values were investigated at 540 nm by an ELISA reader. Cell viability(%) is the result of dividing the absorption rate of each sample by that of the control (untreated L929 cells) [41,42].

### 2.12. Animal study

An in vivo wound-healing study was conducted on 18 male Wistar rats (200–250 g). Under sterile conditions, the animals were divided into six different groups (three in each group), including Sterile gauze as the negative control, a commercial dressing as the positive control, and the synthesized NC films. All procedures were conducted under ethical approval (IR.TUMS.REC.1395.2848) and in line with US NIH guidelines [43,44]. The wound area was quantified by the ImajeJ software and calculated using the following Equation (4):

(4) $$\mathrm{Wound}\;\mathrm{closure}(\%)=[A_{0}–A_{t})/A_{0}]\times 100$$

Where A_0_ is the initial area of the created wound, and A_t_ is the wound area at time t [45,46].

### 2.13. Histological study

The rats were sacrificed on day 14 using CO_2_ gas asphyxiation (according to AVMA guidelines for the euthanasia of animals) [47,48]. Tissue specimens were collected, and 5 µm sections were prepared and stained with hematoxylin and eosin (H&E). Next, the stained tissue sections were photographed and evaluated using an optical microscope [49,50].

### 2.14. Statistical analysis

One-way ANOVA was used to evaluate differences among treatment groups. Data are reported as mean ± standard deviation (SD) from three independent replicates (*n* = 3). Differences were considered statistically significant at *p* < 0.05.

## 3. Results and discussion

### 3.1. Structural characterization by ATR-FTIR spectroscopy

**Figure 1.**
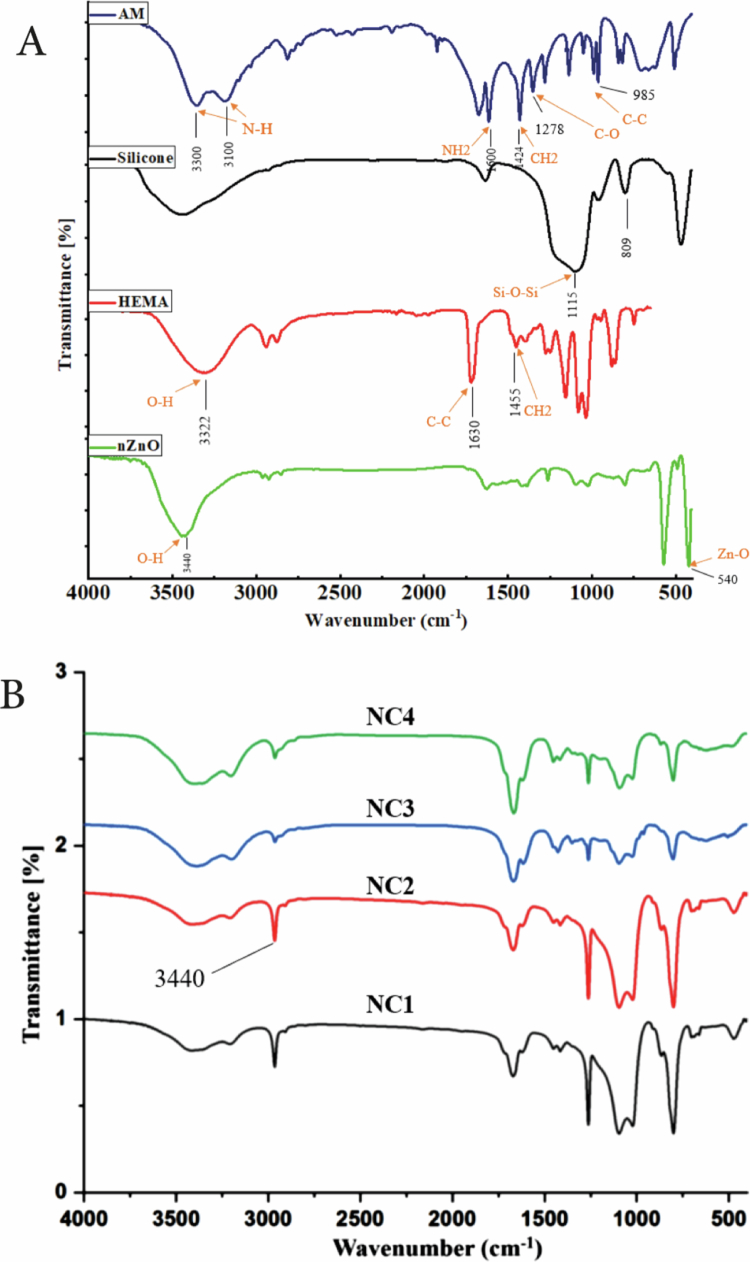
(A) ATR-FTIR spectra of silicone, HEMA, AAm monomers, and ZnO NPs. (B) ATR-FTIR spectra of synthesized nanoparticles.

ATR-FTIR spectra of AAm, HEMA, silicone, and ZnO NPs are shown in Figure 1A and B shows poly (HEMA-co-AAm)/silicone nanoparticles in four ratios. Figure 1A displays the spectrum of ZnO NPs. The Zn─O stretching and hydroxyl groups of pure ZnO NPs are responsible for two characteristic absorption peaks at 540 and 3440 cm^−1^, respectively [51]. In the spectrum of AAm in Figure 1A, two peaks in the range of 3300 and 3100 cm^−1^ are related to the asymmetric stretching and the symmetric stretching of the N─H group. Also, the deep absorption peak at 1600 cm^−1^ is related to the amide group; the peak at 1278 cm^−1^ refers to C─O stretching, and the peak at 1424 cm^−1^ refers to CH_2_ stretching. The C═C stretching vibration peak is also at 985 cm^−1^ [52]. The broad absorption peak at 3000–3500 cm^−1^ in the HEMA spectrum is related to the vibration of OH bonds. Also, peaks in the range of 1550 and 1630 cm^−1^ correspond to the vibrations of the CH_2_ and CC bonds, respectively. The appearance of peaks at around 1600, 1090, and 800 cm^−1^ can be noted for silicone spectra in Figure 1A. Liu et al. reported that silicone has an acute peak at 1562 cm^−1^ that is characteristic of the Si-CH_3_ bond as well as the large double peaks characteristic of Si─O─Si bonds at 1100 and 1018 cm^−1^ [53]. Similarly, in Figure 1A, a deep peak in the range of 1115 cm^−1^ is observed in the spectrum of silicone. Figure 1B displays the spectra of synthetic nanoparticles. From the comparison of Figure 1A and B, AAm, HEMA, and silicone support the successful incorporation of these components into the polymer network. The absence of multiple peaks of AAm monomer in the range of 900 to 1500 cm^−1^, Figure 1B shows that HEMA-AAm copolymer is formed [54]. A broad band near ~3400 cm^−1^ is also observed, which is commonly attributed to O─H stretching, consistent with the hydrophilic component of the IPN structure.

### 3.2. Investigating the size of synthesized IPN nanoparticles

**Figure 2.**
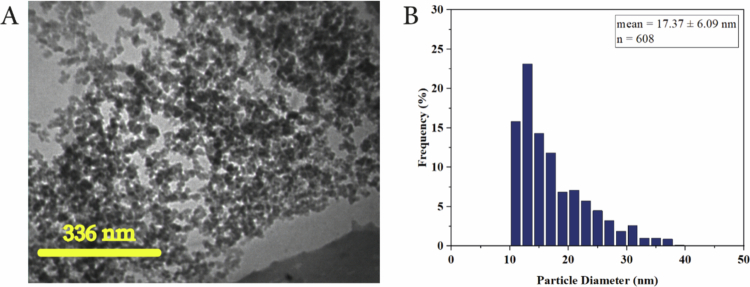
(A) Transmission electron microscopy (TEM) of Poly(HEMA-co-AAm)/silicone IPN nanoparticles, scale bar: 336 nm. (B) TEM particle size distribution of Poly(HEMA-co-AAm)/silicone IPN nanoparticles measured by ImageJ (*n* = 608, bin width = 2 nm). Mean particle diameter = 17.37 ± 6.09 nm.

TEM images (Figure 2) show a sample of Poly(HEMA-co-AAm)/silicone IPN nanoparticles with particle sizes of 17.37 ± 6.09 nm. According to Figure 2A and B, the particles appear to be evenly distributed across the field, with minimal aggregation and consistent morphology. To enhance the robustness of the TEM analysis, a particle size distribution histogram was incorporated, thereby improving the statistical reliability of the results. This suggests a well-controlled synthesis process, likely resulting in desirable mechanical and biological properties. In similar studies, IPNs with size distributions showing less than 10% deviation are considered highly uniform; this sample appears to fall within that range [55]. In addition, most particles exhibit near-spherical shapes with smooth edges and no sharp angles. Such morphology typically results from surface tension during particle formation and contributes to better suspension stability and enhanced performance in biological environments [34]. Interestingly, the measured particle size is consistent with the scale bar in the TEM images. This size range is ideal for applications in drug delivery, tissue engineering, and nanocomposites, offering high surface area and efficient cellular interaction [56]. According to related publications, IPNs designed for biomedical use often fall within the 10–100 nm range, placing this sample in a highly favorable zone [57].

### 3.3. Morphology investigations

**Figure 3.**
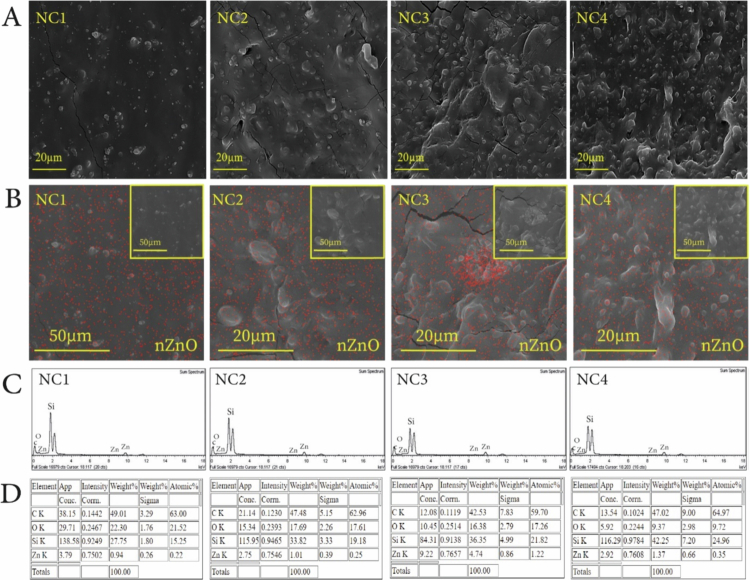
(A) SEM photomicrographs of the cross-section of NC films; (B) EDX images of NC films; (C) EDX Spectra of NC films with different silicone/ZnO concentration; and (D) elemental analysis from EDX of NC Films.

Figure 3A shows the SEM images of NC films. The morphology observed in SEM images can be interpreted by analyzing the distribution and interaction of phases, particularly the continuous phase and the dispersed phase. The SEM images show a rough yet homogeneous surface, indicating a uniform distribution of silicone throughout the samples. Here, silicone acts as the dispersed phase, and its particles are clearly distinguishable in SEM images, often appearing as bright or dark spots. In addition, the presence of the IPN nanoparticle and ZnO has caused bumps on the surface. Specifically, ZnO NPs can act as nucleation sites and generate localized protrusions, thereby influencing the micro- and nano-scale surface features. Previous studies have shown that substrates with ZnO NPs confirm that the presence of nanoparticles increases surface heterogeneity and roughness amplitude [58,59]. It has been reported that a large difference in the degree of homogeneity, hydrophilicity, or miscibility between the two components of the membrane leads to the creation of a regular crystalline phase and a uniform structure [60]. In this regard, hydrophilic (HEMA-co-AAm) and hydrophobic silicone polymers have been combined with silicone nanocomposite films. Wang et al. reported that the existence of a uniform surface indicates a strong connection between the constituent components of the hydrogel [61]. Azizi et al. stated that the addition of inorganic NPs, such as ZnO, creates a rough and uneven surface in the hydrogel [62]. Additionally, SEM-EDX images are used to identify elements and their dispersion in the sample structure [63,64]. The images in Figure 3B illustrate the dispersion of ZnO NPs in the silicone-based hydrogel films obtained by EDX mapping. SEM-EDX area mapping and line scans report Zn content in both wt.% and at.% across multiple regions (*n* ≥ 5), with low spatial variability (Zn = 4.8 ± 0.3 wt.%, CV = 6.2%). These complementary measurements collectively confirm a homogeneous ZnO distribution, as shown in Figure 3C and D.

### 3.4. Hydrophilicity measurement

**Figure 4.**
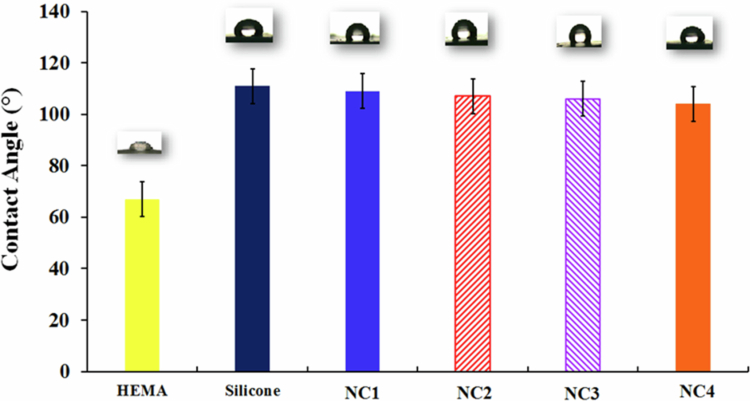
Contact angle measurements of HEMA, silicone, and nanocomposite films containing IPN nanoparticles and ZnO.

The sample's hydrophilic properties were investigated by measuring the water contact angle (WCA) at three different points of the surface. A WCA of less than 90° corresponds to hydrophilic surfaces, and more than 90° corresponds to hydrophobic surfaces [65]. The contact angle results for HEMA, silicone, and all silicone NC films are shown in Figure 4. HEMA with a contact angle of 67° is highly hydrophilic, and pure silicone with a contact angle of 110° is highly hydrophobic. The combination of silicone with all samples results in a contact angle within the hydrophobic range, specifically between 105° and 108°. Hydrophobic surface properties, such as non-adhesion, anti-fouling, and self-cleaning, led to clinical applications of these materials [66–70]. In addition, the presence of ZnO NPs also contributed to increased hydrophobicity in the samples to some extent [71]. According to studies, the hydrophobicity of surfaces depends on the surface morphology and chemical structure of compounds [72–74]. Consistent with these results, Santiago et al. synthesized silicone/ZnO nanocomposite and reported a hydrophobic surface with a contact angle of 117° [71].

### 3.5. Swelling ratio

**Figure 5.**
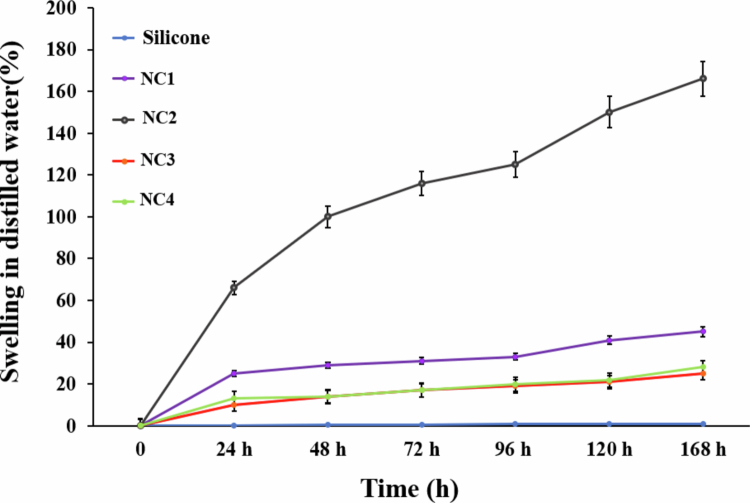
Swelling ratio of nanocomposite films in distilled water.

One of the most effective properties in the functional role of biomaterials is the swelling property [75]. Different factors, such as the hydrophilicity of materials, the crosslinking density, the pH of the aqueous media, the environmental temperature, and the ionic strength, can markedly affect the swelling capacity of hydrogels [76]. The water absorption rate of all silicone-based nanocomposite films was measured for one week and is reported in Figure 5. Swelling measurements were performed in both distilled water and phosphate-buffered saline (PBS), with the results in PBS being nearly comparable (on average ~5% lower) to those in distilled water, thereby confirming the relevance of the chosen conditions to physiological environments. Notably, Silicone is a completely hydrophobic polymer [77], so its swelling rate was about 1%. On the other hand, PHEMA and AAm are hydrophilic polymers, and their impressive swelling properties have been reported individually or in the form of poly (HEMA-co-AAm) in different studies [78–80]. In this study, to overcome the inherent hydrophobicity of silicone, poly(HEMA-co-AAm) segments were successfully incorporated into the matrix. This modification introduces hydrophilic functional groups, significantly improving the water uptake and swelling behavior of the composite [81]. As shown in Figure 5, NC2 exhibited the highest water absorption (165%), whereas NC3 showed the lowest swelling (25%). These results indicate that incorporating poly (HEMA-co-AAm) significantly improves the swelling capacity of silicone-based films, which is a key parameter for wound dressing performance [23]. Therefore, it can be suggested that the addition of poly (HEMA-co-AAm) significantly improves the swelling capacity of the silicone, which is an important parameter for evaluating its applications in wound dressing. Similarly, it has been reported that when the concentration of a hydrophilic polymer, such as Poly(AAm), increases in the IPN powder, a constant increase in the swelling ratio is observed [82]. Also, with the increase of hydrophilic segments in the network, the space between macromolecules decreases, which ultimately leads to an increase in water penetration [83,84].

### 3.6. Gel content

**Figure 6.**
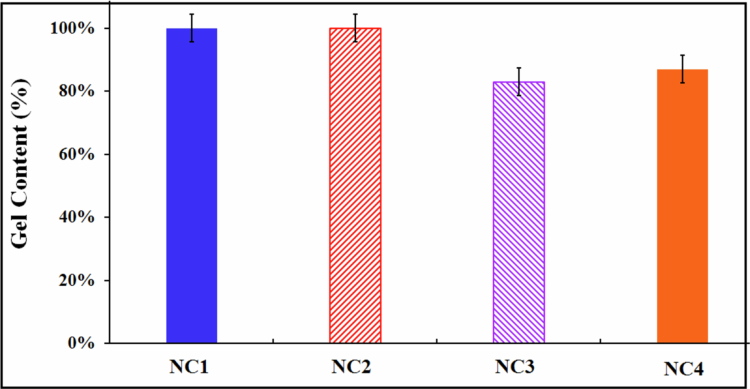
Gel content (%) of the nanocomposite films.

The percentage of gel content in hydrogels indicates the quality of the cross-links and the cohesion of the polymers in the hydrogel [85]. Additionally, the interaction between the components of the hydrogel determines the degree of crosslinking and the hydrogel crystallinity [86,87]. Previous studies have reported that silicone can enhance interactions within composite hydrogel systems, resulting in a higher percentage of gel content [88]. In this case, the ratio of silicone to other components is usually important [89]. Kim et al. reported that the presence of silicone substrate increased the percentage of gel content and the stability of polyethylene glycol methacrylate content in the hydrogel [90]. Similarly, in this study, the percentage of gel content in the silicone-based films decreased by increasing the percentage of poly (HEMA-co-AAm) and decreasing the amount of silicone in the IPN powder. According to Figure 6, NC1 and NC2 films have 100% gel content, whereas NC3 and NC4 have reduced their content by about 25%. The presence of silicone, which contains Si─O─Si units, acts as a physical crosslinker and increases the density of cross-links, which causes the stability and survival of the wound dressing structure [91]. The weaker the polymer chain, the faster the degradation of the structure [92]. The results of the gel content test of some commercial samples have shown that the samples lose a high percentage of the gel content after dissolving in distilled water [93–95]. Moreover, the high percentage of water absorption increases the rate of structural destruction [96]. Researchers reported that increased water content in nanocomposites accelerates degradation of the polymer-nanoparticle matrix, suggesting similar effects on hydrogels [97].

### 3.7. Water vapor transmission rate (WVTR)

**Figure 7.**
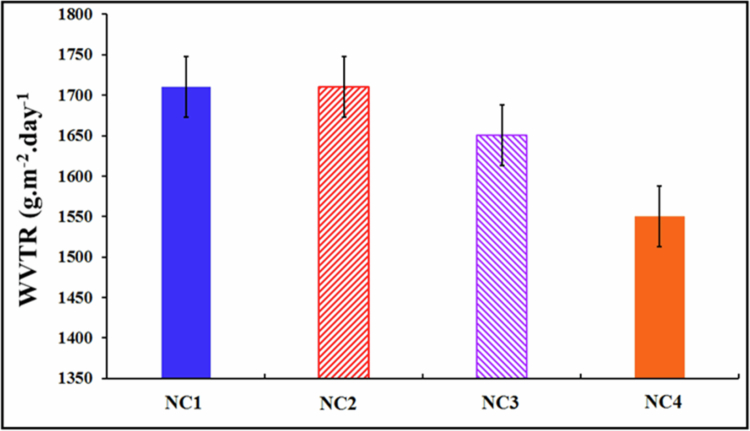
Water vapor transition rate values of the nanocomposite.

An appropriate water vapor transmission rate (WVTR) is essential for an effective wound dressing [98]. A high WVTR causes the formation of scars, and a low rate of WVTR results in the accumulation of exudates and increases bacterial growth [99]. Reported WVTR values for normal skin, skin with a first-degree burn, and granulating wounds are 200, 300, and 5000 g m^−2^ d^−1^, respectively [100]. Therefore, an ideal wound dressing should have a WVTR of about 1500–2500 g m^−2^ d^−1^, which would prevent dryness and the formation of exudates in the wound bed [101]. Figure 7 shows the WVTR values of the prepared films, which were approximately 1700 g m^−2^ d^−1^. According to studies, silicone has a low WVTR rate due to its hydrophobic structure [102–104]. In this regard, Du et al. added polydimethylsiloxane-modified gelatin to silicone, increasing the WVTR to 500 g m^−2^ d^−1^ [104]. Similarly, this study incorporated hydrophilic polymers such as poly(HEMA-co-AAm) into the silicone matrix, increasing WVTR to the desired range. For comparison, the WVTR values for commercial dressings, such as Bioclusive and Tegaderm, are approximately 394 g m^−2^ d^−1^ and 491 g m^−2^ d^−1^, respectively [105,106]. Overall, the synthesized silicone-based films with a WVTR of about 1700 g m^−2^ d^−1^ can be effective in wound healing.

### 3.8. Mechanical investigation

**Figure 8.**
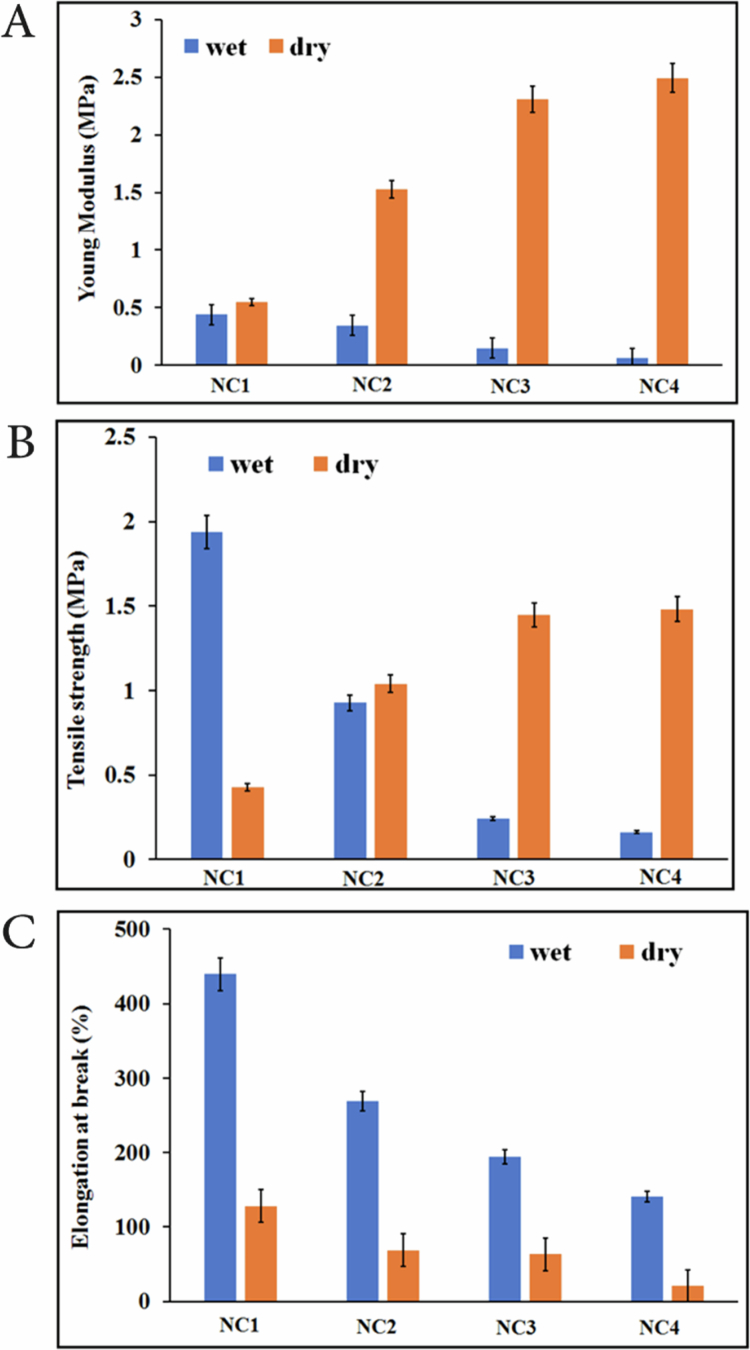
Mechanical properties of nanocomposite films. A: Young modulus, B: Tensile strength, and C: Elongation at break (%) of the samples.

For the best coverage of the wound during the treatment, the wound dressing must be resistant and flexible [107]. The tensile test was performed for all samples (*n* = 3) at ambient temperature with a 10 mm/min tensile rate. Figure 8A, B, and C presents the Young's modulus, tensile strength, and elongation at break of the samples, respectively. According to the results, by increasing the percentage of silicone in the samples, Young's modulus decreased; as a result, the flexibility of the samples increased. This trend is consistent with previous reports and may be attributed to the interpenetration of silicone chains between existing polymers, which increases flexibility [108,109]. Additionally, integrating silicone with the IPN powder improved the mechanical strength of the films [110]. This may be regarded as a more condensed network structure that allows for a more efficient distribution of stress. Researchers have also reported that the presence of NPs enhances the mechanical properties of the nanocomposite by lowering the tension within the polymer network [111]. The dry condition for all samples exhibited a significant rise in mechanical strength and Young's modulus. Another benefit is that it increases the gel structure's rigidity, resulting in greater tensile strength [112]. Specifically, the tensile strengths of NC1 and NC4 increase to 0.42755 ± 0.10415 and 1.48095 ± 0.10945 MPa in a dry state, respectively. Moreover, Young's modulus was higher in the dry state than in the equilibrium swelling state, and the elongation at break was higher in the equilibrium swelling condition compared to the dry phase. This, in turn, causes a decrease in Young's modulus when the film is wet compared to when it is dry. The results show that by decreasing the percentage of silicone in NC4 compared to NC1, the elongation at the breakpoint decreased from 128% to 21% in a dry state and from 440% to 140% in the equilibrium-swollen state. Overall, the tensile strength and elastic modulus data suggest that the prepared NC films provide adequate mechanical properties, enabling them to safely protect the wound region from unexpected injuries. According to other reported wound dressings, like bioartificial collagen-based dermis (*δ* = 2.5 ± 0.1 MPa), asymmetrical PVA/CS (*δ* = 1.7 ± 0.4 MPa), electrospun gelatin mat (*δ* = 2 ± 1 MPa) [113], and PGE/SR membrane (*δ* = 4.9 ± 0.2 MPa), the silicone rubber NC films with *δ* = 2 ± 2.5 MPa have stronger and more flexible performances [114].

### 3.9. In vitro evaluation

**Figure 9.**
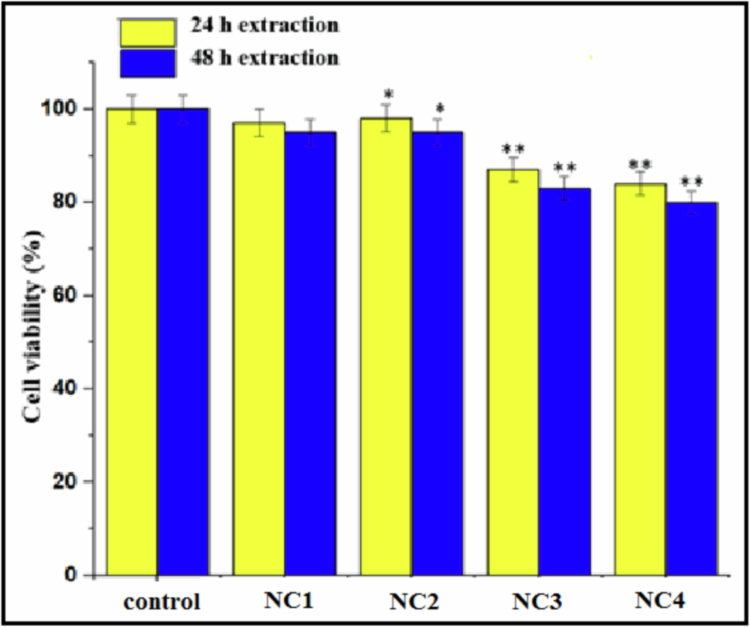
Cell viability of L929 cells treated with NC films extract for 24 hours. Data are presented as mean ± of 3 independent MTT stainings. The variance between the relative sample and control is significant at *p* < 0.05 (*), *p* < 0.01 (**), and *p* < 0.001 (***).

The compounds of the films should be biocompatible [115], minimizing the immune response while maintaining the desired cell adhesion properties [116]. In general, the adhesion of cells on the films indicates their non-toxicity and the biocompatibility of the structure [117]. To evaluate the biocompatibility of all NC films, the MTT test was performed. For this purpose, the extracts were prepared by incubating the samples for 24 and 72 hours, and L929 mouse fibroblast cells were then exposed to these extracts [118]. Cell viability was measured 24 hours after exposure. As shown in Figure 9, the viability of L929 cells was above 95%, which shows that the samples were not toxic. In this regard, the results of the cell migration test in Figure 9 show that the treated cells have the same growth and proliferation capabilities as the control cells. After 24 hours, the cells had grown and migrated, and closed the scratch area. To further support the MTT and migration tests in line with the biocompatibility and non-toxicity of samples, a cell-adhesion assay was also performed to assess surface interactions relevant to biomedical applications [119–121]. Subsequently, it has been reported in various articles that the roughness of the surface causes adhesion and, as a result, the proliferation of cells on the surface [121–123]. These findings are aligned with reports on the biocompatibility of poly(HEMA-co-AAm) hydrogels [124], silicone structures for wound re-epithelialization, and HEMA-based hydrogel structures for wound dressing applications [125,126].

### 3.10. Cell adhesion

**Figure 10.**
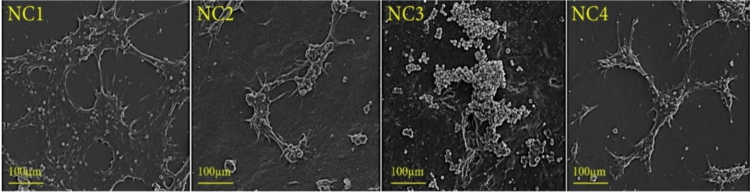
SEM images of mouse L929 cells adhesion on silicone rubber film surfaces.

The SEM images in Figure 10 showed that the L929 cells were attached and survived on the film. The surface roughness of the synthesized silicone rubber NC films likely promoted cell attachment. Subsequently, it has been mentioned in various articles that the roughness of the surface can enhance cell adhesion and subsequently support cell proliferation on biomaterial surfaces [122,123,125]. Panpinit et al. reported that the hydrogel synthesized based on poly (HEMA-co-AAm) by the IPN method was biocompatible [21]. Rui Xu has reported that silicone is biocompatible, and cells can adhere and multiply on it, which results in wound re-epithelization [126]. Also, Massoumi et al. have reported the non-toxicity of silicone coated with HEMA and the suitability of this compound for medical applications.

### 3.11. Antibacterial activities

**Figure 11.**
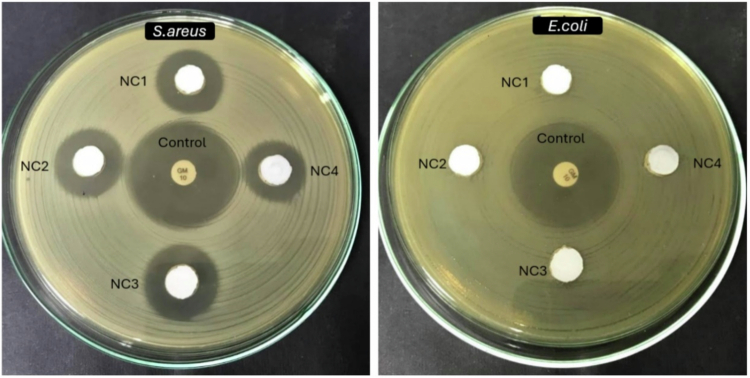
Antibacterial activity of films against *E. coli* and *S. aureus*.

In this study, 1% ZnO NPs were incorporated into all the samples as an antibacterial agent. As shown in Figure 11, samples were cut into discs and placed on agar plates inoculated with *S. aureus* and *E. coli,* alongside an antibiotic disc as a positive control. It should be noted that ZnO NPs have also been used as antibacterial additives in commercial wound dressings. Previous reports suggest that ZnO NPs can damage bacterial cell membranes through the generation of reactive oxygen species (ROS), leading to membrane disruption and bacterial death in both Gram-positive and Gram-negative species. Clear inhibition zones were observed around the synthesized NC films, with diameters of 20 ± 0.63 mm against *s. aureus*. In contrast, no clear bacterial inhibition zone was observed around the synthesized NC films against *E. coli*. It has been mentioned in various studies that silicone-based hydrogel films are resistant to microbial penetration and can be useful in preventing wound infection. It should also be noted that ZnO NPs are predominantly immobilized within the polymer matrix; however, a limited release of Zn^2+^ ions likely occurs under the agar diffusion assay conditions. These ions can diffuse into the surrounding medium and contribute to the observed inhibition zones. In addition, the antibacterial film in our study was prepared with a thickness of less than 0.5 mm, which allowed it to be in direct contact with the bacterial lawn during the agar diffusion assay. Therefore, the antibacterial mechanism involves both contact killing at the surface and ion-mediated diffusion [118].

### 3.12. In vivo investigation

**Figure 12.**
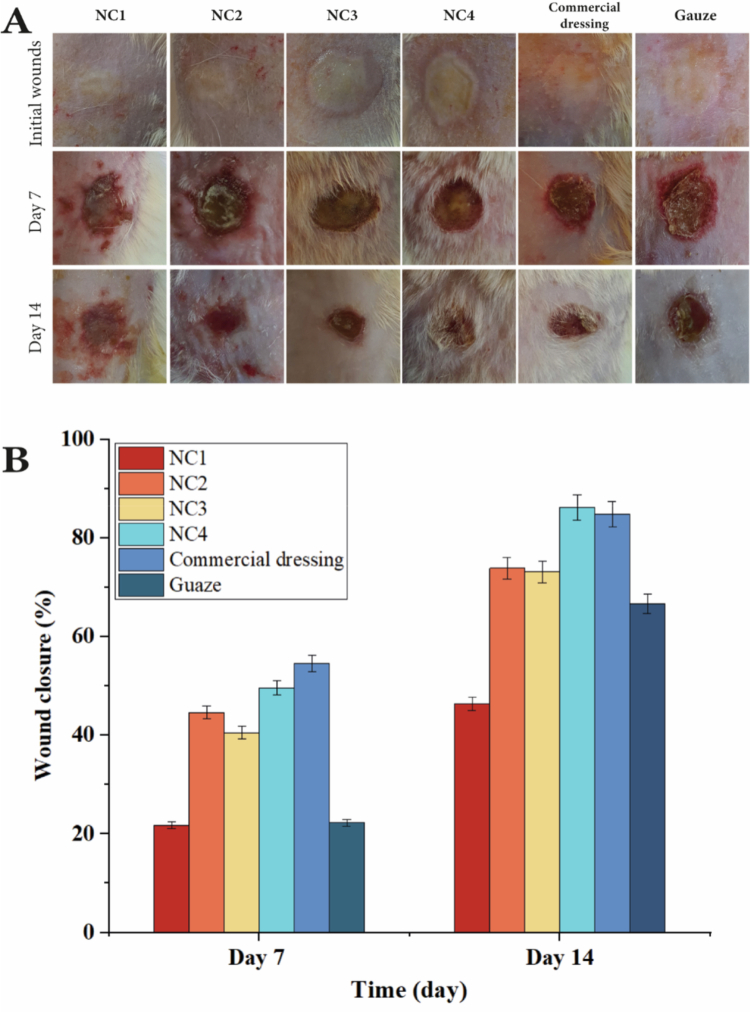
(A) Third-degree burn wound images from treatment of rats on days 0, 7, and 14. The wounds were treated with nanocomposite films, sterile gauze (negative control), and commercial dressings. (B) The wound closure percentages on days 7 and 14 were measured for both the control sample and the treatment group using NC films.

For the macroscopic examination of the wound healing process, NC films, sterile gauze (negative control), and commercial dressing (positive control) were placed on the burn wounds on rats and monitored for 14 days. Figure 12A shows wound healing using the NC films, commercial dressing, and gauze at days 7 and 14 post-treatment. The diameter of the burn wounds that were created was about 10 mm. Histological analysis was performed at the final time point to evaluate healing outcomes, but the tissue sections at this stage still clearly demonstrated the hallmarks of third-degree burns (loss of epidermis, dermis, and adnexal structures), which confirms the initial burn depth. Overall, after 14 days, the NC films improved wound healing compared with both the commercial dressing and gauze, especially NC4. The treated groups did not show an increased inflammatory response, and the NC films may have reduced infection risk by limiting bacterial growth. In addition, wound closure percentages were calculated at each time point, and statistical analyzes were performed to assess differences between groups, as shown in Figure 12B.

### 3.13. Histology evaluation of wound treatment in a third-degree burn skin defect model

**Figure 13.**
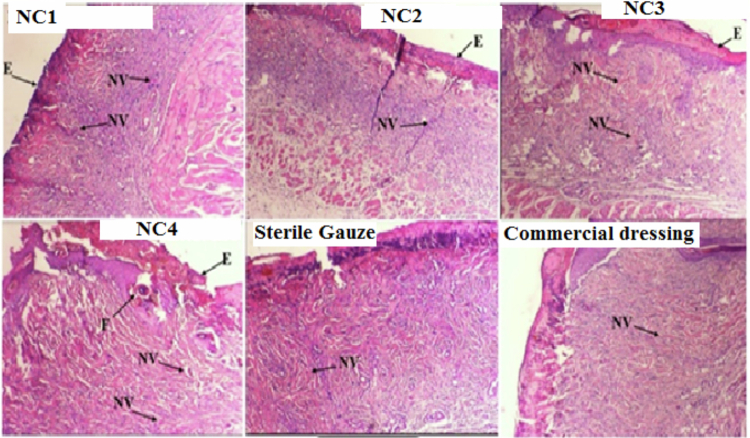
H&E staining image of regeneration skin layers at day 14 after treatment with silicone-based hydrogel film, sterile gauze, and commercial dressings.

Hematoxylin & Eosin (H&E) stained sections of rat burn wound skin collected 14 days after surgery are shown in Figure 13. Microscopic evaluation showed that the wounds treated with NC3 and NC4 exhibited the most advanced healing among the groups. Histological examination indicated that the growth rate of the new epidermis in treated wounds with Silicone-based hydrogel films had increased in the wound area compared to the control group. In addition, a high number of fibroblasts, granulation tissue, and newly formed capillaries were detected in the treated groups, particularly for NC3 and NC4. The presence of fibroblasts is associated with the proliferative phase of wound healing and supports extracellular matrix deposition and tissue remodeling; therefore, increased fibroblast density is indicative of improved healing. The full description of treated wounds with silicone rubber NCs and controls is listed in Table 3. Histological scoring was performed on a 0–4 scale, where 0 indicates non-existence, and 4 indicates the highest value.

**Table 3.** Histopathological findings of wound treatment after 14 days.

Scoring of factors	NC1	NC2	NC3	NC4	Sterile gauze	Commercial dressing
Epithelization	1	1	3	3	0	2
Inflammatory cells	2	2	3	3	2	3
New vessels	2	2	3	3	2	3
Fibroblast	2	2	3	3	2	3
Collagen	2	2	2	2	2	2
All score	9	9	14	14	8	13
Description	Proliferation of fibroblasts and formation of collagens is almost weak to moderate. Necrosis is seen in the wound.	Proliferation of fibroblasts and formation of collagens is almost weak to moderate. Necrosis is seen in the wound.	The epithelium layer is completely joined on both sides of the wound, and some blood vessels can be seen.	The epithelium layer is completely joined on both sides of the wound, and some blood vessels can be seen.	The epithelium layer is not developed, and necrosis is seen on the wound. The amount of inflammatory cells is high and blood vessels is low.	The epithelium layer is not completely connected on both sides of the wound, and necrosis is seen in the middle. The number of blood vessels is also low.

## 4. Conclusion

In this study, a novel nanocomposite was successfully developed by overcoming the thermodynamic incompatibility between hydrophilic and hydrophobic networks. Poly (HEMA‑co‑AAm)/silicone was synthesized in powder form via an IPN polymerization method. The resulting powders were then blended with excess silicone and 1 wt% ZnO nanoparticles to produce uniform silicone‑based nanocomposite films. This innovative structural design synergistically combined the excellent exudate management of the hydrophilic phase with the robust and non-adherent nature of silicone. Based on chemical, mechanical, physical, and in vitro cytocompatibility evaluations, the prepared silicone NC films demonstrate strong potential as wound dressings for burn injuries. Cytotoxicity assessment showed that all materials were non‑toxic to L929 mouse fibroblast cells. In vivo experiments further revealed that all silicone NC films, particularly the 10% silicone/ZnO formulation, enhanced wound repair compared with sterile gauze and a commercial dressing. Overall, the prepared silicone NC films exhibit high performance, favorable mechanical properties, and excellent cell compatibility, supporting their potential application as burn wound dressings.

## Data Availability

Data available on request from the authors.
